# Type I and III interferons disrupt lung epithelial repair during recovery from viral infection

**DOI:** 10.1126/science.abc2061

**Published:** 2020-06-11

**Authors:** Jack Major, Stefania Crotta, Miriam Llorian, Teresa M. McCabe, Hans Henrik Gad, Simon L. Priestnall, Rune Hartmann, Andreas Wack

**Affiliations:** 1 Immunoregulation Laboratory, The Francis Crick Institute, London, UK.; 2 Bioinformatics and Biostatistics, The Francis Crick Institute, London, UK.; 3 Department of Molecular Biology and Genetics, Aarhus University, Aarhus, Denmark.; 4 Department of Pathobiology and Population Sciences, The Royal Veterinary College, Hatfield, UK.; 5 Experimental Histopathology Science Technology Platform, The Francis Crick Institute, London, UK.

## Abstract

Excessive cytokine signaling frequently exacerbates lung tissue damage during respiratory viral infection. Type I (IFN-α and IFN-β) and III (IFN-λ) interferons are host-produced antiviral cytokines. Prolonged IFN-α and IFN-β responses can lead to harmful proinflammatory effects, whereas IFN-λ mainly signals in epithelia, thereby inducing localized antiviral immunity. In this work, we show that IFN signaling interferes with lung repair during influenza recovery in mice, with IFN-λ driving these effects most potently. IFN-induced protein p53 directly reduces epithelial proliferation and differentiation, which increases disease severity and susceptibility to bacterial superinfections. Thus, excessive or prolonged IFN production aggravates viral infection by impairing lung epithelial regeneration. Timing and duration are therefore critical parameters of endogenous IFN action and should be considered carefully for IFN therapeutic strategies against viral infections such as influenza and coronavirus disease 2019 (COVID-19).

## Interferons interfere with lung repair

Interferons (IFNs) are central to antiviral immunity. Viral recognition elicits IFN production, which in turn triggers the transcription of IFN-stimulated genes (ISGs), which engage in various antiviral functions. Type I IFNs (IFN-α and IFN-β) are widely expressed and can result in immunopathology during viral infections. By contrast, type III IFN (IFN-λ) responses are primarily restricted to mucosal surfaces and are thought to confer antiviral protection without driving damaging proinflammatory responses. Accordingly, IFN-λ has been proposed as a therapeutic in coronavirus disease 2019 (COVID-19) and other such viral respiratory diseases (see the Perspective by Grajales-Reyes and Colonna). Broggi *et al.* report that COVID-19 patient morbidity correlates with the high expression of type I and III IFNs in the lung. Furthermore, IFN-λ secreted by dendritic cells in the lungs of mice exposed to synthetic viral RNA causes damage to the lung epithelium, which increases susceptibility to lethal bacterial superinfections. Similarly, using a mouse model of influenza infection, Major *et al.* found that IFN signaling (especially IFN-λ) hampers lung repair by inducing p53 and inhibiting epithelial proliferation and differentiation. Complicating this picture, Hadjadj *et al.* observed that peripheral blood immune cells from severe and critical COVID-19 patients have diminished type I IFN and enhanced proinflammatory interleukin-6– and tumor necrosis factor-α–fueled responses. This suggests that in contrast to local production, systemic production of IFNs may be beneficial. The results of this trio of studies suggest that the location, timing, and duration of IFN exposure are critical parameters underlying the success or failure of therapeutics for viral respiratory infections.

*Science*, this issue p. 706, p. 712, p. 718; see also p. 626

## Teaser

Interferons can both enhance and abate the severity of respiratory viral infections.

During infection with respiratory viruses, disease severity is linked to lung epithelial destruction, owing to both cytopathic viral effects and immune-mediated damage. Epithelial loss contributes to acute respiratory distress syndrome, pneumonia, and increased susceptibility to bacterial superinfections. Thus, the restoration of damaged epithelial tissues is paramount to maintain lung function and barrier protection.

Interferons (IFNs) are key to the antiviral host defense. IFN-α and IFN-β (IFN-α/β) and IFN-λ are induced upon viral recognition, and they trigger transcription of IFN-stimulated genes with antiviral functions in infected and bystander cells. Because of widespread expression of the type I IFN receptor (IFNAR) in immune cells, IFN-α/β responses can result in immunopathology during viral infections, including influenza virus and severe acute respiratory syndrome–coronavirus 1 (SARS-CoV-1) (*1*–*4*). The IFN-λ receptor (IFNLR) is mainly expressed at epithelial barriers, and IFN-λ responses are therefore often characterized by their ability to confer localized antiviral protection at the site of infection without driving damaging proinflammatory responses like those associated with IFN-α/β. In addition to antiviral and proinflammatory activity, IFNs exert antiproliferative and proapoptotic functions (*5*). Despite a growing understanding of immunopathology in respiratory viral infection, it is unknown how IFN responses affect lung epithelial repair.

**Fig. 1:**
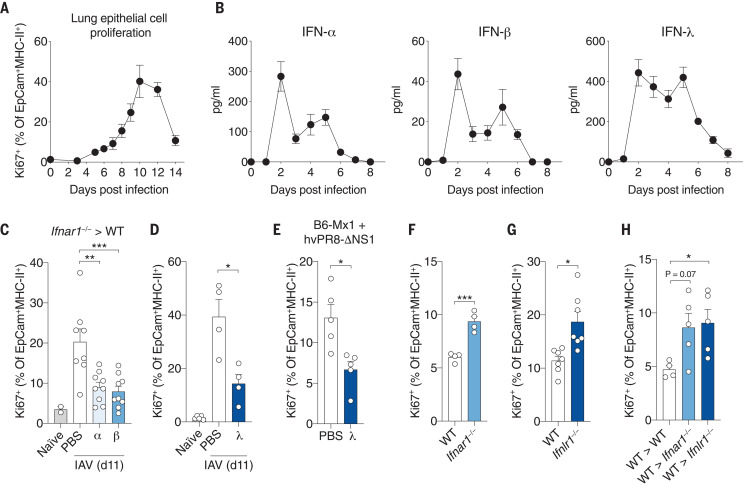
Type I and III IFNs reduce epithelial cell proliferation during lung repair. (**A** and **B**) Mice were infected intranasally with 10^4^ TCID_50_ X31 (H3N2) influenza virus in 30 μl. (A) Proliferating (Ki67^+^) AT2 cells (EpCam^+^MHCII^+^CD49f^lo^) were measured by flow cytometry (*n* = 5 mice). (B) Type I and III IFN levels were detected in BALF (*n* = 4) on indicated days after infection. (**C** and **D**) X31-infected mice were administered IFNs every 24 hours (on days 7 to 10 after infection). Proliferating (Ki67^+^) AT2 cells (EpCam^+^MHCII^+^CD49f^lo^) were measured by flow cytometry on day 11 after infection. (C) Lethally irradiated WT mice were injected with *Ifnar1*^−/−^ BM cells. After reconstitution, influenza virus–infected chimeric mice were treated with phosphate-buffered saline (PBS) control (*n* = 8), IFN-α (*n* = 9), or IFN-β (*n* = 9). Naïve controls were uninfected, untreated BM chimeric mice (*n* = 2). (D) Infected WT mice were treated with IFN-λ (*n* = 4) or PBS control (*n* = 4). Naïve controls were uninfected, untreated WT mice (*n* = 5). IAV, influenza A virus. (**E**) B6-Mx1 mice were infected with 2.5 × 10^3^ TCID_50_ hvPR8-ΔNS1 (H1N1) and treated with IFN-λ (*n* = 4) or PBS control (*n* = 4). IFN treatment and lung analysis were performed as for (C) and (D). (**F** to **H**) Lungs from X31-infected WT mice (*n* = 4 to 7), *Ifnar1*^−/−^ mice (*n* = 4) (F), *Ifnlr1*^−/−^ mice (*n* = 7) (G), and BM chimeric mice (*n* = 4 to 5) (H) were harvested; and proliferating (Ki67^+^) AT2 cells were measured by flow cytometry on day 8 after infection. All data are representative of at least two independent experiments. Data are shown as means ± SEM, and statistical significance was assessed by one-way analysis of variance (ANOVA) with Dunnett’s posttest [(C), (D), and (H)] or unpaired two-tailed Student’s *t* test [(E) to (G)]. *P* > 0.05; *P ≤ 0.05; **P ≤ 0.01; ***P ≤ 0.001.

Influenza virus infection in C57BL/6 (B6) wild-type (WT) mice resulted in weight loss accompanied by substantial immune cell infiltration and lung damage (fig. S1, A to D). Recovery from infection coincided with the onset of epithelial regeneration (fig. S1, B to D). To further investigate the dynamics of lung repair after influenza virus infection, epithelial cell proliferation was analyzed by flow cytometry using the proliferation marker Ki67 (see gating strategy in fig. S2). During steady-state conditions, type II alveolar epithelial (AT2) cells (EpCam^+^MHCII^+^CD49f^lo^) (*6*–*8*) showed a low rate of turnover (Fig. 1A). However, after influenza virus–induced lung damage, AT2 cells underwent rapid proliferation starting at days 5 to 7 after infection, which correlated with mouse recovery and weight gain (Fig. 1A and fig. S1B).

To compare the dynamics of epithelial recovery with IFN production, we analyzed IFN subtypes (IFN-α, IFN-β, and IFN-λ) in bronchoalveolar lavage fluid (BALF) throughout infection. IFNs were produced rapidly, peaking 2 days after infection (Fig. 1B). The magnitude of IFN-λ production was significantly greater than that of IFN-α/β, both in duration and in length of peak production. Notably, only IFN-λ was detected 7 to 8 days after infection, coinciding with the onset of epithelial recovery (Fig. 1, A and B). Thus, after influenza virus infection, signaling triggered by IFNs—particularly by IFN-λ—overlaps with the onset of lung repair.

To compare the effects of equipotent amounts of IFN-α, IFN-β, and IFN-λ on lung repair, mice were treated during recovery from influenza virus infection (7 to 10 days after infection; figs. S3A and S4). To study the effects of IFN treatment specifically on epithelial cells, we generated irradiation bone marrow (BM) chimeras in which WT recipients were given *Ifnar1*^−/−^ BM cells (*Ifnar1*^−/−^ → WT BM chimeric), thereby restricting IFNAR expression to the stromal compartment.

In chimeric mice, both IFN-α and IFN-β treatments significantly reduced the proliferation of AT2 cells on day 11 after influenza virus infection (Fig. 1C). Similarly, IFN-λ treatment reduced AT2 cell proliferation in WT mice (Fig. 1D). Reductions in proliferation were independent of changes in viral burden (fig. S3, B and C). The IFN-λ–mediated reduction in AT2 cell proliferation did not require IFN-λ signaling in neutrophils (*9*–*11*), as neutrophil depletion in WT mice using an anti-Ly6G monoclonal antibody had no effect (fig. S3, D and E). A caveat to bear in mind when using inbred mouse strains for influenza virus infection is their lack of a functional Mx1 protein, a crucial IFN-inducible influenza virus restriction factor in both mice and humans (*12*). We therefore infected mice expressing functional Mx1 alleles (B6-Mx1) with the influenza virus strain hvPR8-ΔNS1 for a more–clinically relevant influenza model. IFN-λ treatment significantly reduced epithelial proliferation in the presence of functional Mx1 as well (Fig. 1E).

We next used *Ifnar1*^−/−^ and *Ifnlr1*^−/−^ mice to determine the role of endogenous IFNs during lung repair. AT2 cells were analyzed on day 8 after influenza virus infection, the time when IFN signaling and epithelial cell proliferation overlapped (Fig. 1, A and B). Both *Ifnar1*^−/−^ and *Ifnlr1*^−/−^ mice had improved AT2 cell proliferation compared with WT controls (Fig. 1, F and G). This was dependent on IFN signaling specifically through the epithelium, because receptor deficiency in the stromal compartment alone was sufficient to increase lung epithelial cell proliferation (Fig. 1H). Improved proliferation was independent of major changes in viral burden (fig. S5A). Viral control in individual IFN receptor–knockout mice was likely unaffected owing to redundancy between type I and III IFN antiviral responses in epithelial cells (*13*, *14*). Despite type I and III IFN redundancy in viral control (fig. S5A), the lack of redundancy in antiproliferative IFN responses—with both *Ifnar1*^−/−^ and *Ifnlr1*^−/−^ mice displaying enhanced epithelial proliferation (Fig. 1, F to H)—led us to further interrogate the phenotype. IFNAR signaling has been previously shown to be important for the production of IFN-λ during influenza virus infection (*15*, *16*). Consistent with these findings, we observed a significant reduction in IFN-λ (and in IFN-α/β) production in *Ifnar1*^−/−^ mice compared with WT; yet, we saw little change in IFN-α/β levels in *Ifnlr1*^−/−^ mice (fig. S5B). Thus, the improved epithelial proliferation in *Ifnar1*^−/−^ mice may result from reduced IFN-λ. IFN production defects in *Ifnar1*^−/−^ mice are linked to reduced steady-state priming in the absence of tonic IFNAR activation in immune cells (*17*). To circumvent this, we administered an anti-IFNAR monoclonal antibody (MAR1-5A3) only from the onset of influenza virus infection. Anti-IFNAR treatment maintained steady-state priming required for IFN-λ production (fig. S5C), despite blocking IFN-α/β signaling through IFNAR (fig. S5D). Notably, anti-IFNAR treatment from day 0 or day 3 after infection had no effect on lung epithelial cell proliferation (fig. S5E). Thus, in murine influenza virus infection, endogenous IFN-λ responses are most effective in disrupting epithelial regeneration during influenza recovery through direct effects on epithelial cells.

**Fig. 2:**
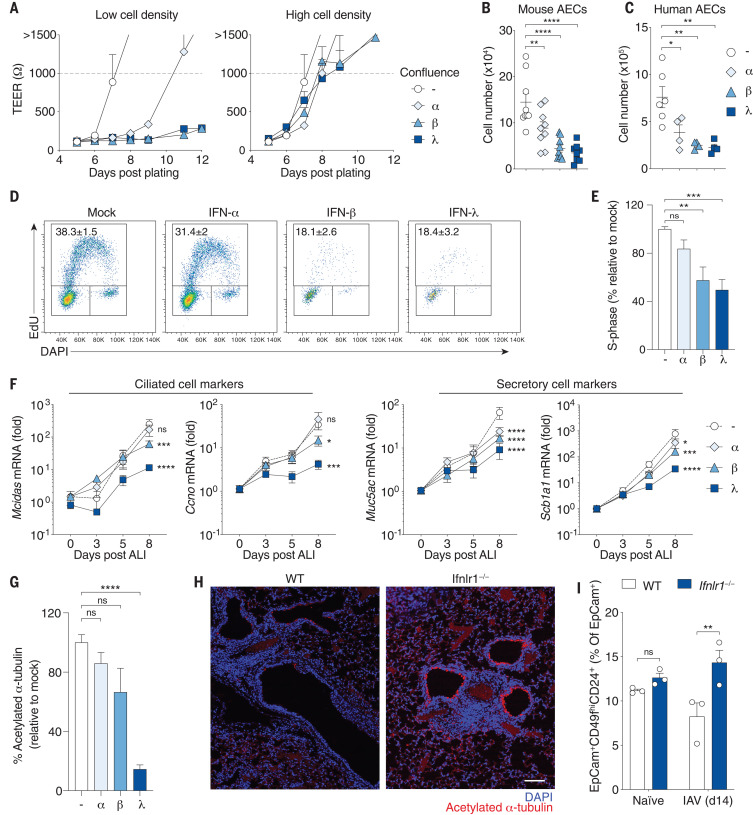
IFN signaling blocks AEC growth and differentiation. (**A**) Murine AECs were seeded at a low density (500 cells per transwell) or high density (10^4^ cells per transwell) in the presence of equivalent doses of IFN-α, IFN-β, IFN-λ, or media control, and then grown for 12 days (*n* = 3 transwells for all conditions). Confluence was determined by measuring transepithelial electrical resistance (TEER) (>1000 ohm = confluent cultures). (**B**, **D**, and **E**) Proliferating murine AEC cultures (2 days before exposure to an ALI) were treated for 5 days with IFNs (2 days before ALI to day 3 after ALI), and effects on growth were determined by cell number (*n* = 9) (B) and incorporation of the thymidine analog EdU to measure proliferation (*n* = 9) [(D) and (E)]. DAPI, 4′,6-diamidino-2-phenylindole; EdU, 5-ethynyl-2′-deoxyuridine. (**C**) Primary human AEC cultures were treated with IFNs for 5 days and cells were counted (*n* = 4 to 6). (**F** and **G**) Murine AECs were grown to confluence, then exposed to an ALI for 2 days. IFNs were then administrated for 6 days during ALI exposure (*n* = 6 for all conditions). Differentiation was determined by mRNA expression of the indicated genes (F) and the level of acetylated α-tubulin staining in cultures (G). (**H** and **I**) WT and *Ifnlr1*^−/−^ mice were infected with influenza virus, and lungs were analyzed by immunofluorescence (DAPI or acetylated α-tubulin) on day 10 after infection (*n* = 4 mice) (H), and flow cytometry (EpCam^+^CD49f^hi^CD24^+^) on day 14 after infection (*n* = 3) (I). All data are representative of at least three independent experiments. Data are shown as means ± SEM, and statistical significance was assessed by one-way [(B), (C), (E), and (G)] or two-way [(F) and (I)] ANOVA with Dunnett’s posttest. Scale bar represents 100 μm (H). ns, not significant; *P* > 0.05; *P ≤ 0.05; **P ≤ 0.01; ***P ≤ 0.001; ****P ≤ 0.0001.

To understand mechanistically how IFNs exert the observed antiproliferative effects, we set up primary murine airway epithelial cell (AEC) cultures. AECs undergo rapid proliferation and differentiation upon exposure to an air-liquid interface (ALI), which recapitulates lung repair processes observed in vivo (*18*, *19*). IFNs used for in vitro assays were titrated on AEC cultures to compare IFN subtypes at equivalent biological potencies (fig. S6). All three IFN subtypes significantly impaired the growth of AEC cultures, with IFN-β and IFN-λ having the most significant effects (Fig. 2, A to E, and fig. S7A). Similar effects were observed when primary human AEC cultures were treated with equivalent doses of IFN subtypes (Fig. 2C). Growth inhibitory effects were dependent on the presence of the respective IFN receptor (fig. S7B). IFN-β or IFN-λ treatment increased the frequency of apoptotic or necrotic cells (defined as annexin V^+^ and TO-PRO-3^+^) (fig. S7, C and D); however, the growth inhibitory effects of IFNs were only observed in actively dividing cultures (fig. S7, E to G). Thus, the increase in apoptosis observed may occur as a result of failed progression through the cell cycle after IFN treatment, as has been seen previously (*20*).

We next examined the effects of IFNs on AEC differentiation. After acute damage, populations of basal cells and Scgb1a1^+^ secretory cells give rise to secretory and multiciliated cell subtypes (*21*). To study the effects of IFNs on AEC differentiation, we initiated IFN treatment late during the course of AEC growth, during air exposure when AEC differentiation is induced (fig. S8A). IFN-β and IFN-λ treatment significantly reduced the expression of genes pertaining to multiciliated (*Mcidas* and *Ccno*) and secretory (*Muc5AC* and *Scb1a1*) cell differentiation (Fig. 2F). Expression of the basal cell marker *Krt5* remained unchanged or was increased by IFN-λ treatment, which suggests maintenance of stemness (fig. S8B). We also found reduced numbers of multiciliated cells in AEC cultures (acetylated α-tubulin^+^) after IFN-λ treatment, but we did not observe this with IFN-α or IFN-β treatment (Fig. 2G and fig. S8C). In vivo, *Ifnlr1^−/−^* mice displayed increased multiciliated cells in repairing conducting airways on day 10 after influenza virus infection (Fig. 2H). Using flow cytometry, we quantified this increase in the frequency of differentiated AECs (EpCam^hi^CD49f^hi^CD24^+^) composed of multiciliated, goblet, and club cells (Fig. 2I and fig. S2) (*22*). Thus, IFN-λ signaling reduces the capacity for basal cell differentiation during recovery from influenza virus infection.

**Fig. 3:**
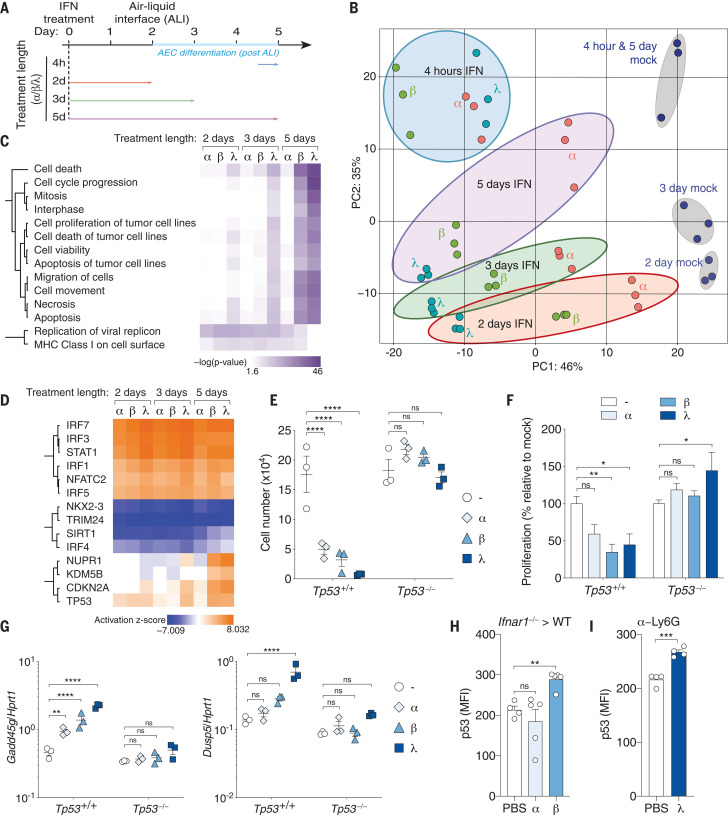
Type I and III IFNs activate antiproliferative and cell death pathways in AECs via induction of p53. (**A**) Schematic diagram for IFN treatment of murine AECs for RNA-sequencing analysis. (**B**) PCA plot of RNA-sequencing data from AECs after IFN treatment and from untreated controls. (**C**) Heatmap for significant differences in canonical pathways for nine pairwise comparisons between indicated IFN treatment and the respective mock, at each time point (fold change >1.5, one-way ANOVA with Benjamini-Hochberg correction, *P* < 0.05). Gene expression was compared using ingenuity pathway comparison analysis. MHC, major histocompatibility complex. (**D**) Predicted upstream transcriptional regulators of differentially expressed genes (ingenuity pathway analysis). (**E** to **G**) WT and *p53*^−/−^ murine AECs were treated with IFN subtypes for 5 days and measured for growth by cell number (E), CFSE (carboxyfluorescein diacetate succinimidyl ester) dilution (F), and mRNA expression of indicated genes (G) (*n* = 3 transwells for all conditions). (**H** and **I**) *Ifnar1*^−/−^ → WT BM chimeric mice (*n* = 4 to 5 mice) (H) and α-Ly6G treated mice (*n* = 4) (I) infected with influenza virus (X31), and treated with IFN every 24 hours consecutively for 4 days (days 7 to 10 after infection), before EpCam^+^MHCII^+^CD49f^lo^ AT2 cells were analyzed for p53 mean fluorescence intensity (MFI) on day 11 after infection by flow cytometry. All data are representative of at least two independent experiments [(E) to (I)]. Data are shown as means ± SEM, and statistical significance was assessed by two-way [(E) to (G)] or one-way (H) ANOVA with Dunnett’s posttest or by unpaired two-tailed Student’s *t* test (I). ns, not significant; *P* > 0.05; *P ≤ 0.05; **P ≤ 0.01; ***P ≤ 0.001; ****P ≤ 0.0001.

To understand how IFNs mediate antiproliferative effects, we performed RNA sequencing on IFN-treated AEC cultures (Fig. 3A). Principal components analysis (PCA) clustered 4-hour IFN-treated samples together regardless of subtype (Fig. 3B), which confirmed equal subtype dosage on the basis of previous titrations (fig. S9A). Five days of IFN-β or IFN-λ treatment clustered AECs together separately from untreated controls on both PC1 and PC2 (Fig. 3B). Gene ontology analysis confirmed that genes contributing to this variance are involved in IFN signaling and epithelial cell development (supplementary text and fig. S9B). Ingenuity pathway analysis revealed induction of pathways regulating cell cycle and cell death after prolonged IFN treatment, most significantly induced by IFN-λ across all time points (Fig. 3C). Predicted upstream transcriptional regulators identified typical regulators of IFN function—including STAT (signal transducer and activator of transcription) and IRF (IFN regulatory factor) proteins—in addition to cell cycle regulators (Fig. 3D). We identified the tumor suppressor protein p53 as a top candidate regulating IFN-inducible antiproliferative effects. p53 has previously been shown to directly regulate IFN-α/β antitumor responses (*23*). Gene set enrichment analysis (GSEA) identified IFN-mediated induction of the p53 pathway (fig. S9C), and we identified induction of p53-regulated downstream targets in expression data (fig. S9D). To confirm the role of p53, we utilized *Tp53*^−/−^ AEC cultures. IFN-mediated reduction in AEC growth, differentiation, and induction of antiproliferative downstream p53 target genes *Gadd45g* and *Dusp5* (*24*, *25*) was p53-dependent, with no changes observed in *Tp53*^−/−^ AECs (Fig. 3, E to G, and fig. S9E). We next examined whether IFNs regulate p53 activity in epithelial cells during lung repair in vivo. To study IFN effects specifically in the lung epithelium, we once again generated *Ifnar1*^−/−^ → WT BM chimeric mice for IFN-α or IFN-β treatment, and we depleted neutrophils in WT mice with anti-Ly6G for IFN-λ treatment (fig. S3A). IFN-β and IFN-λ, but not IFN-α, significantly up-regulated p53 expression in repairing lung epithelial cells (Fig. 3, H and I). Thus, IFN-β and IFN-λ mediate antiproliferative effects in AECs via the induction of p53.

**Fig. 4:**
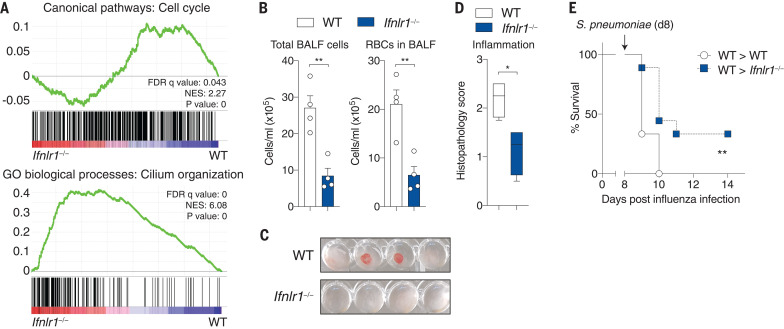
*Ifnlr1*^−/−^ mice have improved lung repair, reduced damage, and improved epithelial barrier function. WT and *Ifnlr1*^−/−^ mice were infected with 10^4^ TCID_50_ X31 influenza virus (X31). (**A**) GSEA plots of RNA-sequencing datasets from WT or *Ifnlr1*^−/−^ bulk lung epithelial cells (EpCam^+^) on day 8 after infection. FDR, false discovery rate; NES, normalized enrichment score; GO, gene ontology. (**B** and **C**) Total cell and red blood cell (RBC) (TER-119^+^) number in BALF on day 8 after infection (*n* = 4 mice for both WT and *Ifnlr1*^−/−^). (**D**) Histopathological analysis of hematoxylin and eosin (H&E) lung sections on day 9 after infection (*n* = 4 for both WT and *Ifnlr1*^−/−^). (**E**) Lethally irradiated WT and *Ifnlr1*^−/−^ mice were injected with WT BM cells. After reconstitution, chimeric mice were challenged with 2 × 10^5^ colony-forming units TIGR4 in 30 μl on day 8 (d8) after influenza virus infection (*n* = 8 WT; *n* = 9 *Ifnlr1*^−/−^). All data are representative of at least two independent experiments [(B) to (E)]. Data are shown as means ± SEM, and statistical significance was assessed by unpaired two-tailed Student’s *t* test (B), Mann-Whitney *U* test (D), or log-rank (Mantel-Cox) test (E). *P ≤ 0.05; **P ≤ 0.01.

Our data support a key role for IFN signaling, particularly IFN-λ, in the reduction of epithelial proliferation and differentiation during lung repair. We therefore tested whether IFNs alter the state or barrier function of lung epithelia. RNA sequencing of sorted lung epithelial cells (EpCam^+^CD31^−^CD45^−^) from influenza virus–infected WT or *Ifnlr1*^−/−^ mice confirmed an up-regulation of pathways pertaining to proliferation and multiciliogenesis in *Ifnlr1*^−/−^ mice (Fig. 4A). Improved repair correlated with reduced lung damage, with a reduction in both the total number of cells and the number of red blood cells in the BALF of *Ifnlr1*^−/−^ mice at day 8 after infection (Fig. 4, B and C, and fig. S10A). Additionally, *Ifnlr1*^−/−^ mice had fewer immune cells infiltrating lung tissue (Fig. 4D). In humans, influenza virus–induced epithelial damage increases susceptibility to infection by opportunistic bacterial pathogens, including *Streptococcus pneumoniae* (*26*). To measure the effects of IFN-λ on lung barrier function, we challenged influenza virus–infected mice with *S. pneumoniae*. Both full-IFNLR-knockout mice and mice lacking IFNLR in the stromal compartment (WT → *Ifnlr1*^−/−^) had improved survival after bacterial superinfection (Fig. 4E and fig. S10B). Thus, IFN-λ signaling reduces the capacity for epithelial repair, which results in prolonged lung damage, compromised barrier function, and increased susceptibility to bacterial superinfection.

In this work, we describe a mechanism by which type I and III IFN signaling aggravates lung pathology during respiratory viral infection. Although all three IFN subtypes reduced lung proliferation after treatment during influenza recovery, only endogenous IFN-λ compromised repair. This is likely because of increased IFN-λ production during infection combined with greater induction of antiproliferative pathways. A recent study has shown that IFN-λ produced by dendritic cells inhibits lung epithelial repair after viral recognition (*27*). Influenza virus–infected macaques have been found to have an elevated IFN signature late during infection in bronchial tissue (*28*). Additionally, COVID-19 patients have displayed strong induction of IFN and p53 signaling in collected BALF samples (*29*). Analysis of lung tissue and BALF from respiratory virus–infected patients experiencing severe disease will provide insight into the mechanisms regulating disease pathogenesis. IFN-λ treatment early during influenza virus infection is protective in mice, offering antiviral protection without the proinflammatory responses associated with IFN-α/β (*30*, *31*). By studying specific effects in the respiratory epithelium, we identified a mechanism by which IFN exacerbates respiratory virus disease, independent of immunomodulation. Our data indicate the need for effective regulation of host IFN responses and the importance of timing and duration when considering IFNs as therapeutic strategies to treat respiratory virus infections. Optimal protection could be achieved by strong induction of IFN-stimulated genes early during infection to curb viral replication followed by timely down-regulation of IFN responses, thereby enabling efficient lung epithelial repair.

## Supplementary Materials

science.sciencemag.org/content/369/6504/712/suppl/DC1 Materials and Methods Supplementary Text Figs. S1 to S10 Table S1 References (*32*–*38*) MDAR Reproducibility Checklist Data S1 to S3

## Appendix

View/request a protocol for this paper from *Bio-protocol*.
